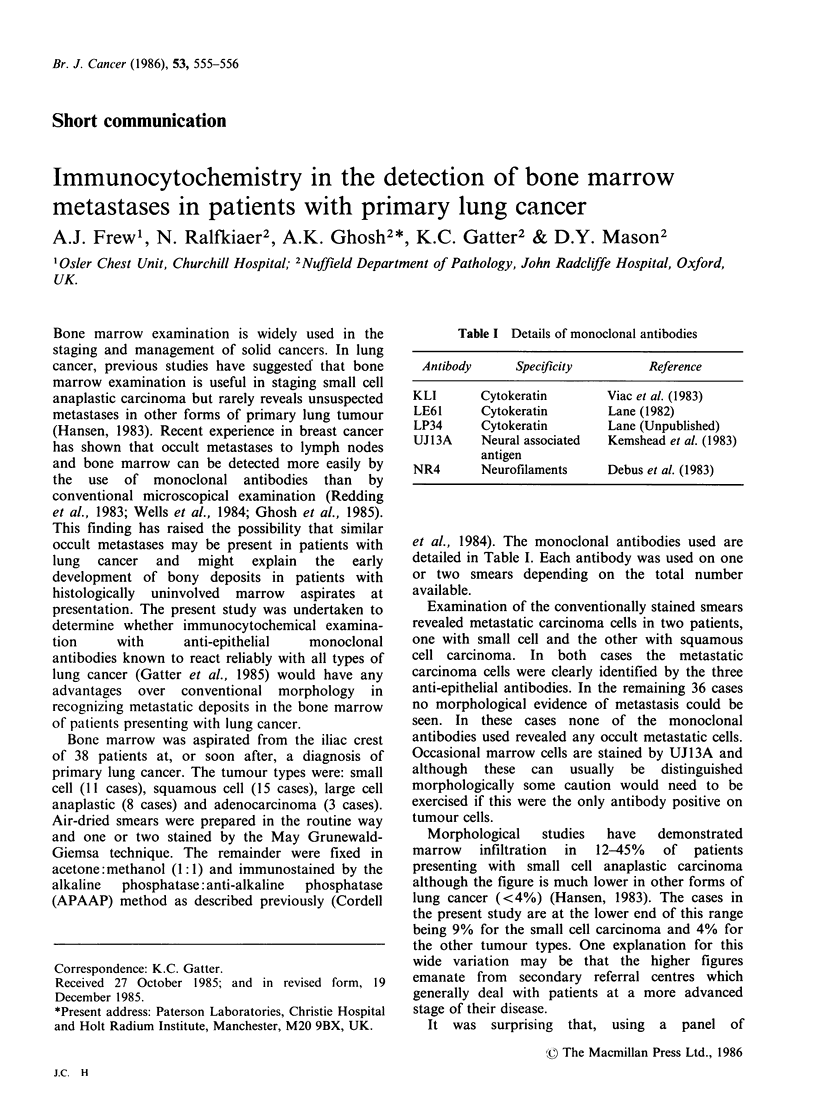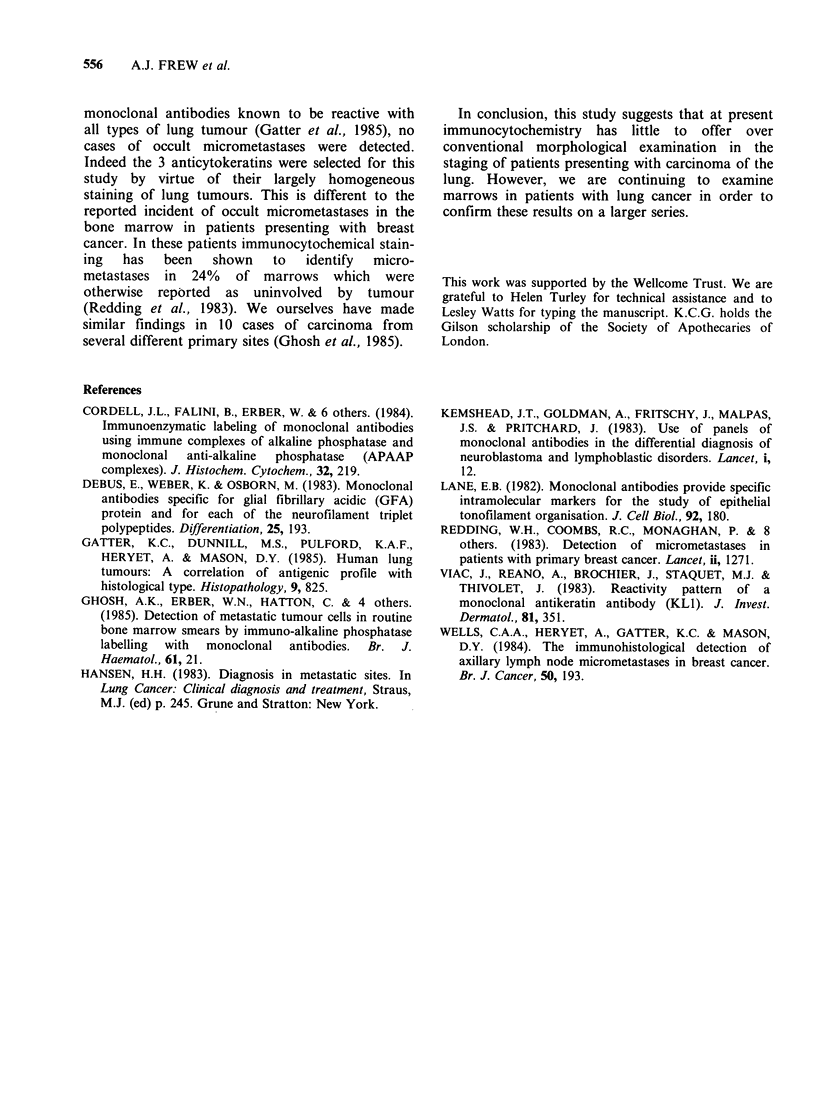# Immunocytochemistry in the detection of bone marrow metastases in patients with primary lung cancer.

**DOI:** 10.1038/bjc.1986.86

**Published:** 1986-04

**Authors:** A. J. Frew, N. Ralfkiaer, A. K. Ghosh, K. C. Gatter, D. Y. Mason


Br. J. Cancer (1986), 53, 555–556

### Short communication

### Immunocytochemistry in the detection of bone marrow metastases in patients with primary lung cancer

A.J. Frew[1], N. Ralfkiaer[2], A.K. Ghosh[2]*, K.C. Gatter[2] & D.Y. Mason[2]

[1]*Osler Chest Unit, Churchill Hospital;* [2]*Nuffield Department of Pathology, John Radcliffe Hospital, Oxford, UK.*

Bone marrow examination is widely used in the staging and management of solid cancers. In lung cancer, previous studies have suggested that bone marrow examination is useful in staging small cell anaplastic carcinoma but rarely reveals unsuspected metastases in other forms of primary lung tumour (Hansen, 1983). Recent experience in breast cancer has shown that occult metastases to lymph nodes and bone marrow can be detected more easily by the use of monoclonal antibodies than by conventional microscopical examination (Redding *et al.*, 1983; Wells *et al.*, 1984; Ghosh *et al.*, 1985). This finding has raised the possibility that similar occult metastases may be present in patients with lung cancer and might explain the early development of bony deposits in patients with histologically uninvolved marrow aspirates at presentation. The present study was undertaken to determine whether immunocytochemical examination with anti-epithelial monoclonal antibodies known to react reliably with all types of lung cancer (Gatter *et al.*, 1985) would have any advantages over conventional morphology in recognizing metastatic deposits in the bone marrow of patients presenting with lung cancer.

Bone marrow was aspirated from the iliac crest of 38 patients at, or soon after, a diagnosis of primary lung cancer. The tumour types were: small cell (11 cases), squamous cell (15 cases), large cell anaplastic (8 cases) and adenocarcinoma (3 cases). Air-dried smears were prepared in the routine way and one or two stained by the May Grunewald-Giemsa technique. The remainder were fixed in acetone:methanol (1:1) and immunostained by the alkaline phosphatase:anti-alkaline phosphatase (APAAP) method as described previously (Cordell *et al.*, 1984). The monoclonal antibodies used are detailed in Table I. Each antibody was used on one or two smears depending on the total number available.

**Table I** Details of monoclonal antibodies

| *Antibody* | *Specificity* | *Reference* |
|---|---|---|
| KLI | Cytokeratin | Viac *et al.* (1983) |
| LE61 | Cytokeratin | Lane (1982) |
| LP34 | Cytokeratin | Lane (Unpublished) |
| UJ13A | Neural associated antigen | Kemshead *et al.* (1983) |
| NR4 | Neurofilaments | Debus *et al.* (1983) |

Examination of the conventionally stained smears revealed metastatic carcinoma cells in two patients, one with small cell and the other with squamous cell carcinoma. In both cases the metastatic carcinoma cells were clearly identified by the three anti-epithelial antibodies. In the remaining 36 cases no morphological evidence of metastasis could be seen. In these cases none of the monoclonal antibodies used revealed any occult metastatic cells. Occasional marrow cells are stained by UJ13A and although these can usually be distinguished morphologically some caution would need to be exercised if this were the only antibody positive on tumour cells.

Morphological studies have demonstrated marrow infiltration in 12–45% of patients presenting with small cell anaplastic carcinoma although the figure is much lower in other forms of lung cancer (<4%) (Hansen, 1983). The cases in the present study are at the lower end of this range being 9% for the small cell carcinoma and 4% for the other tumour types. One explanation for this wide variation may be that the higher figures emanate from secondary referral centres which generally deal with patients at a more advanced stage of their disease.

It was surprising that, using a panel of

Correspondence: K.C. Gatter.
Received 27 October 1985; and in revised form, 19 December 1985.
*Present address: Paterson Laboratories, Christie Hospital and Holt Radium Institute, Manchester, M20 9BX, UK.

© The Macmillan Press Ltd., 1986

monoclonal antibodies known to be reactive with all types of lung tumour (Gatter *et al.*, 1985), no cases of occult micrometastases were detected. Indeed the 3 anticytokeratins were selected for this study by virtue of their largely homogeneous staining of lung tumours. This is different to the reported incident of occult micrometastases in the bone marrow in patients presenting with breast cancer. In these patients immunocytochemical staining has been shown to identify micrometastases in 24% of marrows which were otherwise reported as uninvolved by tumour (Redding *et al.*, 1983). We ourselves have made similar findings in 10 cases of carcinoma from several different primary sites (Ghosh *et al.*, 1985).

In conclusion, this study suggests that at present immunocytochemistry has little to offer over conventional morphological examination in the staging of patients presenting with carcinoma of the lung. However, we are continuing to examine marrows in patients with lung cancer in order to confirm these results on a larger series.

This work was supported by the Wellcome Trust. We are grateful to Helen Turley for technical assistance and to Lesley Watts for typing the manuscript. K.C.G. holds the Gilson scholarship of the Society of Apothecaries of London.

### References

CORDELL, J.L., FALINI, B., ERBER, W. & 6 others. (1984). Immunoenzymatic labeling of monoclonal antibodies using immune complexes of alkaline phosphatase and monoclonal anti-alkaline phosphatase (APAAP complexes). *J. Histochem. Cytochem.*, **32,** 219.

DEBUS, E., WEBER, K. & OSBORN, M. (1983). Monoclonal antibodies specific for glial fibrillary acidic (GFA) protein and for each of the neurofilament triplet polypeptides. *Differentiation*, **25,** 193.

GATTER, K.C., DUNNILL, M.S., PULFORD, K.A.F., HERYET, A. & MASON, D.Y. (1985). Human lung tumours: A correlation of antigenic profile with histological type. *Histopathology*, **9,** 825.

GHOSH, A.K., ERBER, W.N., HATTON, C. & 4 others. (1985). Detection of metastatic tumour cells in routine bone marrow smears by immuno-alkaline phosphatase labelling with monoclonal antibodies. *Br. J. Haematol.*, **61,** 21.

HANSEN, H.H. (1983). Diagnosis in metastatic sites. In *Lung Cancer: Clinical diagnosis and treatment*, Straus, M.J. (ed) p. 245. Grune and Stratton: New York.

KEMSHEAD, J.T., GOLDMAN, A., FRITSCHY, J., MALPAS, J.S. & PRITCHARD, J. (1983). Use of panels of monoclonal antibodies in the differential diagnosis of neuroblastoma and lymphoblastic disorders. *Lancet*, **i,** 12.

LANE, E.B. (1982). Monoclonal antibodies provide specific intramolecular markers for the study of epithelial tonofilament organisation. *J. Cell Biol.*, **92,** 180.

REDDING, W.H., COOMBS, R.C., MONAGHAN, P. & 8 others. (1983). Detection of micrometastases in patients with primary breast cancer. *Lancet*, **ii,** 1271.

VIAC, J., REANO, A., BROCHIER, J., STAQUET, M.J. & THIVOLET, J. (1983). Reactivity pattern of a monoclonal antikeratin antibody (KL1). *J. Invest. Dermatol.*, **81,** 351.

WELLS, C.A.A., HERYET, A., GATTER, K.C. & MASON, D.Y. (1984). The immunohistological detection of axillary lymph node micrometastases in breast cancer. *Br. J. Cancer*, **50,** 193.